# Supplementary material for: Diagnosis-Specific Work Disability before and after Lumbar Spine Decompression Surgery—A Register Study from Sweden
Source: Int J Environ Res Public Health. 2021 Aug 25;18(17):8937. doi: 10.3390/ijerph18178937 (PMC8430561; doi:10.3390/ijerph18178937)
Supplement: Supplementary file 1 [file ijerph-18-08937-s001.zip › ijerph-1266398-supplementary.pdf]

Supplementary Table 1. Long-term sickness absence (LTSA) (> 90 net sickness absence days) and disability pension (DP) due to certain diagnoses (ICD-10 codes)[1] in the three years following cohort entry date (t+1, t+2, t+3), among Swedish residents, aged 19-60 years, with low back pain (LBP) treated by lumber spine decompression surgery (LSDS) during 2008-10, with LBP without LSDS and persons with no diagnosed LBP

**LSDS** n (%) 10 800 (100%)		**LBP no LSDS** n (%) 109 179 (100%)		**No diagnosed LBP** n (%) 472 191 (100%)	
**LTSA**	**DP**	**LTSA**	**DP**	**LTSA**	**DP**
**Neoplasm (C00-D48)***					
60 (0.56)	6 (0.06)	745 (0.68)	144 (0.13)	3254 (0.70)	277 (0.06)
**Mental disorders (F00-F99)***					
321 (2.97)	117 (1.08)	4714 (4.32)	1708 (1.56)	13140 (2.78)	3498 (0.74)
**Diseases of the nervous system (G00-G99)***					
57 (0.53)	40 (0.37)	570 (0.52)	307 (0.28)	1192 (0.25)	519 (0.11)
**Cardiovascular diseases (I00-I99)***					
49 (0.45)	15 (0.14)	607 (0.56)	212 (0.19)	2216 (0.47)	609 (0.13)
**Musculoskeletal diseases (M00-M99)***					
1773 (16.42)	402 (3.72)	10 633 (9.74)	2961 (2.71)	8399 (1.78)	1073 (0.23)
**Symptoms, signs and abnormal clinical laboratory findings (R00-R99)***					
55 (0.51)	21 (0.19)	772 (0.71)	224 (0.21)	1155 (0.24)	128 (0.03)
**Injuries/poisoning (S00-T98)***					
119 (1.10)	48 (0.44)	1424 (1.30)	570 (0.52)	3722 (0.79)	579 (0.12)
**Other diagnoses**					
112 (1.04)	32 (0.30)	1777 (1.63)	363 (0.33)	4395 (0.93)	715 (0.15)
**Total**					
**2546 (23.57)**	**681 (6.31)**	**21 242 (19.46)**	**6489 (5.94)**	**37 473 (7.94)**	**7938 (1.68)**

*Significant between group differences tested by Chi2 at level of p <0.0001.
[1]International Statistical Classification of Diseases and Related Health Problems, 10th revision
